# Public telesurveillance service for frail elderly living at home, outcomes and cost evolution: a quasi experimental design with two follow-ups

**DOI:** 10.1186/1477-7525-4-41

**Published:** 2006-07-07

**Authors:** Claude Vincent, Daniel Reinharz, Isabelle Deaudelin, Mathieu Garceau, Lise R Talbot

**Affiliations:** 1 Department of rehabilitation, Laval University, Pavillon Ferdinand-Vandry, Quebec City (Quebec), G1K7P4, Canada; 2 Center of Interdisciplinary Research in Rehabilitation & Social Integration (CIRRIS), Quebec City, Institut de réadaptation en déficience physique de Québec, 525 bvld Wilfrid-Hamel east, Quebec City, Quebec, G1M 2S8, Canada; 3 Department of Preventive and Social medicine, Laval University, Pavillon de l'est, Québec City (Quebec), G1K 7P4, Canada; 4 Department of Nursing, Faculty of Medicine and Health Sciences, Sherbrooke University, 3001, 12^th^avenue, Sherbrooke (Quebec), Canada

## Abstract

**Background:**

Telesurveillance is a technologically based modality that allows the surveillance of patients in the natural setting, mainly home. It is based on communication technologies to relay information between a patient and a central call center where services are coordinated. Different types of telesurveillance systems have been implemented, some being staffed with non-health professionals and others with health professional, mainly nurses. Up to now, only telesurveillance services staffed with non-health professionals have been shown to be effective and efficient. The objective of this study was to document outcomes and cost evolution of a nurse-staffed telesurveillance system for frail elderly living at home.

**Methods:**

A quasi experimental design over a nine-month period was done. Patients (n = 38) and caregivers (n = 38) were selected by health professionals from two local community health centers. To be eligible, elders had to be over 65, live at home with a permanent physical, slight cognitive or motor disability or both and have a close relative (the caregiver) willing to participate to the study. These disabilities had to hinder the accomplishment of daily life activities deemed essential to continue living at home safely. Three data sources were used: patient files, telesurveillance center's quarterly reports and personal questionnaires (Modified Mini-Mental State, Functional Autonomy Measurement System, Life Event Checklist, SF-12, Life-H, Quebec User Evaluation of Satisfaction with Assistive Technology, Caregiver Burden). The telesurveillance technology permitted, among various functionalities, bi-directional communication (speaker-receiver) between the patient and the response center.

**Results:**

A total of 957 calls for 38 registered clients over a 6-month period was recorded. Only 48 (5.0%) of the calls were health-related. No change was reported in the elders' quality of life and daily activity abilities. Satisfaction was very high. Caregivers' psychological burden decreased substantially. On a 3 months period, length of hospital stays dropped from 13 to 4 days, and home care services decreased from 18 to 10 visits/client. Total cost of health and social public services used per client dropped by 17% after the first 3 months and by 39% in the second 3 months.

**Conclusion:**

The ratio of 0.50 calls per client to the call center for health events is three times higher than that reported in the literature. This difference is probably attributable to the fact that nurses rather than non-health professional personnel were available to answer the clients' questions about their health and medications. Cost evolution showed that registering older adults at a telesurveillance center staffed by nurses, upon a health professional recommendation, costs the health care system less and does not have any negative effects on the well-being of the individuals and their families. Telesurveillance for the elderly is effective and efficient.

## Background

Telesurveillance is a telemedicine application that permits to follow patients with medical needs, at home. Telesurveillance is based on communication technologies to put in contact patients with a call center where social and medical services are coordinated [1]. Emergency as well as services provided on a daily base can therefore be offered without the need to institutionalize patients who prefer to stay in their familiar environment [1-3]. In the telemedicine literature, the term "telemonitoring" may involve the transmission of physiological data by the patient online (e.g. blood pressure, glycaemia) and medical supervision or not. When there is no physiological data transmitted by the patient, the term "telesurveillance" is more appropriated [4]. For example, telesurveillance may be helpful for medication, falls, consciousness, home accident, home intrusion, special diet and reminders for different activities.

Different types of telesurveillance services have been devised to support elders at home. The most common is offered by a call center staffed by non health professionals trained to respond to emergencies and to refer cases to a public health information system if necessary. It requires the identification of two designated persons close to the patient, who have agreed to be called in case of an emergency. Another common type of telesurveillance service is offered by a call center staffed by health professionals, mainly nurses. It also requires the identification of persons who can be joined and who have accepted to visit the patient when needed. In this second system, nurses are available 24 hours per day, 7 days a week to answer questions asked by patients, handle emergencies, remind the taking of medication and give instructions about diet. Both types of services require registration of the patients who have to wear a bracelet or medallion personal help button effective over a distance of 30 meters.

Telesurveillance services staffed by non-health professional personnel has been shown to be effective in improving the general quality of life of the elderly, as well various outcomes as anxiety, the feeling of being safe at home, the perception of a positive effect on health [5,6]. It also has been shown to have a positive effect on the elders' functional independence and autonomy in activities of daily living [5,7-11]. The prompt handling of requests for help within an hour has reduced mortality rates [12]. Moreover, the level of satisfaction with the use of telesurveillance services staffed by non-health professional personnel is high satisfactory [[6,13], Rooney, Studenski & Roman in [14,15]]. Also, use of this type of service has a positive effect on the burden on caregivers, especially regarding their level of anxiety about the safety of their family member [6,7,16]. Finally, telesurveillance staffed by non-health professional personnel has been demonstrated to be cost-effective, mainly because it is associated with a reduction of hospitalizations [5,11,17].

Although a clear literature exists on telesurveillance services staffed by non-health professional personnel, up to now, little is known about services to elders at home provided by nurses. It was the aim of this study to evaluate the effectiveness and cost of such a modality.

## Methods

### Design

A quasi-experimental design with two follow-ups was used [18]. Measures were taken before and after the introduction of the telesurveillance service over a 6-month period. No control group was constituted because of ethical concerns. Indeed, community health centers in Quebec have a policy of not denying services deemed to provide a benefit to their patients. Moreover, because of the heterogeneity of the population (see Tables 1 and 3), home environment and the numerous variables that might influence the outcome, pairing patients and caregivers with a control group would have required a sample sizes beyond what could be constituted. To control changes due to contextual elements, we notified them with the Life event checklist [19]. We did not look back more than 3 months before intervention because of the elder and caregiver memory associated to events past three months. Moreover, returning home after hospitalisation maybe a good reason to subscribe to a telesurveillance service. But hospitalisation that happened 4 months ago and more may not be the major reason to subscribe to such a service.

**Table 1:** Characteristics of the older adults and satisfaction with telesurveillance

**Measures n = 38**	**0–3 months before TU^1^**	**0–3 months after TU**	**4–6 months after TU**
**Age (years)**	81.4 (70 to 93)		

**Females (%)**	92.1		

**Diagnoses (%)**			
- musculoskeletal	52.6		
- neurological	36.8		
- cardiovascular	84.2		
- metabolic	60.5		

**Reasons for telesurveillance registration (%)**			
- risks of falls	71.1		
- medication + therapeutic instructions	13.2		
- personal + family responsibilities	7.9		
- anxiety	7.9		

**Cohabitation (%)**			
- alone	65.8		
- with family member	21.1		
- with spouse	13.2		

**Type of dwelling (%)**			
- apartment	42.1		
- house	36.8		
- apartment with services	10.5		
- du/tri/quadri/plex	5.3		
- public housing project	5.3		

**Cognitive impairments (MMMS)^2^**	81.68 ± 12.00	80.89 ± 13.80	82.16 ± 12.61

**Functional autonomy (FAMS)^3^**	-3.04 ± 2.80	-3.01 ± 2.43	-3.73 ± 2.99

**Life Events Checklist^4^**	32.74 ± 0.82	37.45 ± 51.37	34.39 ± 45.53

**TU satisfaction**			
- Technical (QUEST)^5,6^	n.a.	4.59 ± 0.43	4.61 ± 0.39
- Service (QUEST)^5,7^	n.a.	4.75 ± 0.45	4.70 ± 0.42
- Total (QUEST)^5,6,7^	n.a.	4.64 ± 0.39	4.63 ± 0.36
- Telesurveillance satisfaction (added section)^8^	n.a.	4.43 ± 0.58	4.37 ± 0.52

^1^TU = Telesurveillance use.

^2^Norm: 85% for 80–84 year-olds with 5 to 8 years of schooling. See ref [20]. No significant difference, p = 0.613.

^3^Worst score: -87 (very dependent). See ref [22]. No significant difference, p = 0.141.

^4^Norm: Between 0–149 points per year. See ref [19]. No significant difference, p = 0.808.

^5^Scoring: 1 (very dissatisfied) to 5 (very satisfied). See ref [26].

^6^Including: Size, weight, easy to set, safety, solidity, easy to use, comfort, efficiency. See ref [26].

^7^Including: Procedure to get TU, maintenance & repairs, professional and follow-up services. See ref [26].

^8^Including: Medallion, bracelet, emergency button, absent/present function, time to adapt to TU.

### Sample

Patients and caregivers were selected by health professionals from two local community health centers in the Quebec City area. To be eligible, elders had to be over age 65, live at home with a permanent physical, slight cognitive or motor disability or both. These disabilities had to hinder the accomplishment of daily life activities deemed essential to continue living at home safely. Moreover, patients had to have a close caregiver interested in subscribing to the telesurveillance service. Elders who had limitations in using the telesurveillance equipment and answering the questionnaires were excluded (i.e., major cognitive, visual and hearing impairments). Recruitment was made by health professionals who provided home services to the elderly. The target was to recruit 50 elders and their caregivers over a 6-month period. However, due to some lack of motivation by some professionals only 44 elders and their caregivers were recruited and only 38 completed the project over a 6 months period (3 died, 1 was transferred to a seniors' residence and 2 dropped out). None of the elders declined the telesurveillance service when it was offered by their health professional.

### Data sources

Three data sources were used: patients' files (data on home care services provided); telesurveillance center's quarterly reports (utilization data); and personal questionnaires, all validated for the French speaking population. The patients' files were used to document the type and frequency of clinical home care services received (social work, home help, occupational therapy, physiotherapy, nursing care, dietician services, laboratory and medical services). The *telesurveillance center's computerized quarterly report *was used to collect information on reasons for calls, number of calls and accidental calls. Two research assistants were trained to administer the following validated instruments during the home visits at three points of time: 1 week before telesurveillance started, and at 3 and 6 months after the technology was provided. Overall the questionnaires take 90 minutes to 2 hours to fill out:

• *Modified Mini-Mental State (MMMS) *– to detect and estimate the severity of cognitive difficulties. It requires approximately 20 minutes for a qualified health care professional to complete. The maximum score is 100, indicating no deterioration of the cognitive state [20,21].

• *Functional Autonomy Measurement System (FAMS) *– to measure impairments and social roles that cannot be accomplished. It requires a maximum of 40 minutes to complete and measures five dimensions: activities of daily living, mobility, communication, mental functions and household chores. The maximum score is -87, indicating a high dependency [22].

• *Life Events Checklist *– To verify whether contextual elements could have influenced anxiety and to identify whether any additional assistance was provided, the *Social Readjustment Rating Scale *was used [19]. This questionnaire takes five minutes to fill out.

N.B. Frequency and duration of hospitalization were noted through the *Life Events Checklist*, with the confirmation of the caregiver.

• *SF-12 *– This is a generic measure of quality of life which measures eight dimensions: physical function (ADL); role limitations (handicaps) secondary to physical impairments; physical pain; general health, vitality (fatigue and lack of energy); social functioning; limitations due to emotional problems; and mental health (psychological distress and well-being). The questionnaire takes five minutes to fill out [23,24].

• *Life-H *– A standardized questionnaire that measures the level of accomplishment of more than 250 life habits (activities of daily living and social roles). The accomplishment scale ranges from 0 to 9 (0: not performed, 1: performed by a substitute, 2: performed with difficulty and with assistive technology and human assistance, 3: performed with difficulty and with human assistance, ... to 9: performed with no difficulty and no assistance). For the study, a mean score was calculated out of 9 but for only eight life habits relevant to telesurveillance. The instrument also contains a satisfaction scale for each life habit, ranging from 1 to 5. It takes five minutes to fill out this questionnaire [25].

• *QUEST *– The *Quebec User Evaluation of Satisfaction with Assistive Technology *generates a 12-point evaluation of satisfaction related to the use of an assistive device (on a scale from 1 to 5). It also identifies sources of dissatisfaction. This questionnaire requires a maximum of 20 minutes to fill out [26].

• *Caregiver Burden *– A self-administered questionnaire evaluating the burden evaluated on five dimensions: daily living support, preoccupations about well-being, impact on social life, improvement for the care-receiver, improvement for the caregiver, has been filled by caregivers [27,28].

In Quebec, nearly all services consumed by the elders are provided by the public system. The ministry of health was the main source of fees paid to physicians, and for the estimation of unit prices of services provided by other professionals [29]. These unit prices were estimated on the bases of the AS-471 form which collects financial and operational data of each institution majored by the direct allocation method, to take into account support activity centers [30]. Technical units related to laboratory exams where taken from the ministry manual of laboratory medicine [31]. Information on the telesurveillance call center operating costs was obtained from its finance department.

### Characteristics of the telesurveillance technology

The telesurveillance technology used in this study is characterized by its bi-directional communication capabilities (speaker-receiver) between the patient and the calling center. It consists of a telephone and a small battery-powered wireless emergency call transmitter. The wireless transmitter, which can be worn as a medallion or bracelet, is impact and water-resistant, and can be used to place emergency calls, or to answer the phone when the user is not near the base set. The telephone is equipped with oversized, illuminated buttons and a light ergonomic handset, which is compatible with hearing aids. This model possesses many other special features including bi-directional communication up to 30 meters from the phone, and voice reminders (e.g., for medication, catheters, glycaemia, special diet, prescribed exercises, medical appointments, important social functions, daily activity tasks) that can be set for specific times (daily, weekly or only once). Six reminders can be stored simultaneously and programmed remotely. The elder can also answer and speak on the telephone remotely simply by pressing the emergency button, without picking up the phone. Another available function is the ability to get the time, day and date by pressing a button.

### Analysis

To verify if there were any differences before and after intervention (0, 3 and 6 months), ANOVAs were done on the test scores presenting categorical data with a uniform distribution (MMMS, FAMS, SF-12, Life-H, QUEST, Burden). Wilcoxon tests were applied to the Life Events Checklist score, the number of home care services and the number of calls made to the call center (continuous data with a non-normal distribution). Finally, Mann-Whitney tests were applied to the hospitalization data, which came from independent samples (different "n"s at different times).

For the cost analysis, only running costs were considered for two reasons. First, the basic infrastructure was already functional for many years before the experiment. The project has simply introduced some upgrading of an operational calling center. Then, it was supposed that start up costs would be poorly representative of start costs in another setting, considering that further spread of the service would be a replication, hence less costly than an innovation. Moreover, there were no real additional costs for enrolling patients, as their evaluation was part of the current tasks of the heath care professionals (see Table 5). The costs do not include the evaluation of patients and caregivers for the research project; it was performed by a research assistant and was not part of a regular assessment of patient.

## Results

### Participants profile

The majority of patients registered within the telesurveillance center were women. The average age was 81. They had a high rate of cardiovascular problems and where particularly at risk of falling. Most of them lived alone in an apartment (see Table 1). The cohort presented cognitive functions and a functional autonomy within normal ranges throughout the study, with no deterioration observed. Also, based on the information collected on the Life Events Checklist, no major events seemed to have occurred that could have influenced the elders' lives and biased the evaluation of the telesurveillance intervention (see Table 1). The quality of life score was high at the outset but within the norms of the reference group (see notes 1–2 in Table 2); with no significant difference between measurement times. Of the eight daily life activities measured, two were on the average performed with some difficulty (physical activities and community activities); older adults also report that they were "somewhat satisfied" with the performance of those two ADL. However, the score did not change between measurement times (see Table 2). The data on satisfaction were positive. In the second period of 3 months of use, the technology (4.61 out of 5) and the service (4.70 out of 5) were rated "very satisfactory" (Table 1). Comments collected for the purpose of improving the service indicated a lack of familiarity with the vocal reminders and other phone functions as well as with the emergency button (too sensitive, unattractive appearance).

**Table 2:** Impact of telesurveillance on older adults: quality of life and life habits

**Measures (n = 38)**	**0–3 months before TU^1^**	**0–3 months after TU**	**4–6 months after TU**
**Quality of life (SF12)**			
- physical score^2^	35.68 ± 10.78	35.78 ± 8.81	34.87 ± 8.50
- mental score^3^	48.60 ± 10.47	49.26 ± 10.44	49.30 ± 10.33

**Life habits (LIFE-H)**			
Performance per ADL^4^			
1. Food choice	8.67 ± 0.20	8.10 ± 0.48	8.32 ± 0.30
2. Food preparation	7.58 ± 0.52	6.50 ± 0.69	6.73 ± 0.68
3. Sleeping	7.58 ± 0.23	8.05 ± 0.21	8.05 ± 0.21
**4. Physical activities**	**3.96 ± 0.84**	**5.30 ± 0.93**	**4.32 ± 0.84**
5. Going to the toilet	8.17 ± 0.21	8.10 ± 0.32	8.00 ± 0.34
6. Health care	7.54 ± 0.39	7.75 ± 0.47	7.77 ± 0.32
7. Taking on personal responsibilities	6.08 ± 0.59	5.40 ± 0.73	5.41 ± 0.72
**8. Community activities**	**3.25 ± 0.84**	**3.50 ± 0.87**	**2.77 ± 0.78**
Satisfaction with performance of ADL^5^			
1. Food choice	4.13 ± 0.18	3.81 ± 0.25	4.05 ± 0.24
2. Food preparation	4.13 ± 0.17	4.10 ± 0.21	4.52 ± 0.11
3. Sleeping	3.96 ± 0.19	4.05 ± 0.28	4.38 ± 0.16
**4. Physical activities**	**3.29 ± 0.29**	**3.43 ± 0.31**	**3.38 ± 0.29**
5. Going to the toilet	4.17 ± 0.19	4.38 ± 0.11	4.62 ± 0.11
6. Health care	4.33 ± 0.12	4.15 ± 0.17	4.52 ± 0.13
7. Taking on personal responsibilities	4.38 ± 0.13	4.24 ± 0.21	4.57 ± 0.13
**8. Community activities**	**3.92 ± 0.27**	**3.86 ± 0.30**	**3.86 ± 0.27**

^1^TU: Telesurveillance use.

^2^Norm: 38.7 for 75+ years of age. See ref [23].

^3^Norm: 50.0 for 75+ years of age. See ref [23].

^4^2: Performed with difficulty and with assistive technology (AT) and human assistance. 3: Performed with difficulty and with human assistance. 4: Performed with no difficulty and with AT and human assistance. 5: Performed with no difficulty and human assistance. 6: Performed with difficulty and with AT. 7: Performed with difficulty and no assistance. 8: Performed with no difficulty and AT. 9: Performed with no difficulty and no assistance. See ref [25].

^5^3: Somewhat satisfied. 4: satisfied. 5: very satisfied. See ref [25].

The majority of the caregivers were the elders' daughters and most were employed. Table 3 presents a more detailed profile of the participants and their caregivers. Telesurveillance use had a positive and significant impact on three of the five dimensions of the caregiver burden scale. First, thedaily living supportprovided by the caregivers was high on average at the beginning of the study; after 3 months of telesurveillance, there was a significant decrease (p = 0.012). Second, concern for the care recipient's well-being reported by the caregivers was quite high before the introduction of telesurveillance; after 6 months, there was a significant decrease (p = 0.002). Third, caregiver's social life was acceptable in the pre-experimental period. Finally, one can notice a very slight improvement in the caregivers' well-being level after the second 3 months (p = 0.034) (Table 3).

### Use of the telesurveillance service

A total of 957 calls for 38 registered clients over a 6-month period was recorded. Only 48 (5.0%) of the calls were health-related. Calls about technological support dropped from 598 in the first 3 months to 311 in the second period of 3 months use (p = 0.002). Finally, voice reminders (new telesurveillance function) were used only for three elders and were withdrawn at the demand of these patients within the first 3 months. In one case, the patient considered that the objective aimed with the reminder had been met. In the second case, the elder reported a feeling of intrusion by the voice reminders and in the last case, the caregiver reported that the elder was confused. Table 4 presents the details on telesurveillance use.

**Table 3:** Characteristics of the caregivers and impact of telesurveillance on caregiver burden

**Measures (n = 38)**	**0–3 months before TU^1^**	**0–3 months after TU**	**4–6 months after TU**
**Females (%)**	71.0		

**Relationship to elder**			
- Child	76.3		
- Other family member	10.5		
- Spouse	5.3		
- Other (neighbour, friend)	7.9		
- Living with the elder	18.4		
- Working	71.0		

**Impact on caregiver burden**			
- Daily living support^2^	20.53 ± 9.11	18.56 ± 9.61*	19.35 ± 11.10
- Concern for well-being^3^	17.29 ± 4.54	15.92 ± 4.26**	15.63 ± 4.55**
- Impact on social life^4^	42.87 ± 8.81	44.89 ± 8.30	43.60 ± 7.28
- Improvement for the care-receiver^5^	not evaluated	18.33 ± 16.37	22.11 ± 20.70
- Improvement for the caregiver^6^	not evaluated	5.51 ± 6.53	7.65 ± 8.00***

^1^TU: Telesurveillance use.

*Significant difference after 3 months, p = 0.012.

**Significant difference after 3 and 6 months, p = 0.002.

***Significant difference after 6 months, p = 0.034.

^2^Worst score = 57. See ref [27,28].

^3^Worst score = 24. See ref [27,28].

^4^Best score = 51. See ref [27,28].

^5^Best score = 30. See ref [27,28].

^6^Best score = 27. See ref [27,28].

**Table 4:** Use of the public telesurveillance service staffed by nurses

**Measures n = 38**	**0–3 months before TU^1^**	**0–3 months after TU**	**4–6 months after TU**
**Total calls to the center**	**n.a.**	**626**	**331**

**Health (Subtotal)**		**28 (4.5%)**	**20 (5.7%)**

- Emergency (falls)		3	3
- Emergency (cardiac case)		2	0
- Emergency (other)		5	2
- Emergency (follow-up)		11	3
- General questions		7	12

**Technology (Subtotal)**		**598 (95.5%)**	**311 (94.3%)**

- Functioning		23	10
- Error/catch on the emergency button not purposely		309	166*
- Testing		226	61
- Battery & non-urgent calls		40	74
**Reminder functions (nb of clients)**	**n.a.**	**3**	**0**

^1^TU: Telesurveillance use.

*Significant difference after 6 months, p = 0.002

**Table 5:** Impact of telesurveillance on home care interventions and hospitalizations

**Measures (n = 38)**	before TU^1^	after TU		before TU	after TU	
	
	0–3 months	0–3 months	4–6 months	-3-0 months	-3-0 months	4–6 months
**Category of home care worker from the primary care center**	**Number of home visits**			**Cost of home visits^2 ^(CAN$)**		

- Social worker	126	85	62	7207.20	4862.00	3546.40
- Home help	316	280	186	14166.28	12552.40	8338.38
- Occupational/Physiotherapist (106.20$/visit)	79	48	13	8389.80	5097.60	1380.60
- Nurse (60.23$/h)	179	134	130	10781.17	8070.82	7829.90
- Psychologist (200.62$/interview)	8	1	2	1604.96	200.62	401.24
- Dietitian (57.76$/h)	10	19	0	577.60	1097.44	0.00
- Medical services	8	4	7	412.35	220.10	343.40
- Laboratory tests/services	0	3	5	0.00	17.13	28.55

**TOTAL**	726	574	405	43139.36	32118.11	21868.47

**Average/client**	18.4 ± 27.0	15.3 ± 20.1	10.4 ± 16.6*	**1135.25**	**845.21**	**575.49**

**Hospitalisations**				**Average cost of hospitalizations (CAN$)^3^**		

- Admissions	12	10	10			
- Average length of stay (ALS, days)	13	9.5 (-27%)	4 (-58%)			
- Total duration of hospitalizations^4 ^(TDH, days)	155	94 (-39%)	39 (-75%)	62246.75	40781.90	16920.15
- Nb of hospitalized clients	11	6 (-45%)	7 (-36%)			
**Average cost/client (n = 38)**				**1638.07**	**1073.21**	**445.27**
**Cost of operating the telesurveillance center**						
- Cost of the platform (amortized over 5 years)				0.00	1644.75	1644.75
- Maintenance contract				0.00	750.00	750.00
- Equipment purchase cost (estimated at 300 devices/year; 700$/device)				0.00	52500.00	52500.00
- Nurses' salaries^5 ^(for the territory: 5.2 nurses/1200 clients; 24 × 7)				0.00	381953.50	381953.50
- Installation and repair costs (including technician's salary)				0.00	12500.00	12500.00
- Administration				0.00	9000.00	9000.00
**Subtotal**					458348.25	458348.25
**Cost/client (n = 1200)**					**381.96**	**381.96**
**TOTAL**				**2773.32**	**2300.38**	**1402.72**
**Fee paid by the client (25$/month)**					**75.00**	**75.00**

^1^TU: Telesurveillance use.

*Significant difference after 6 months, p = 0.004.

^2^See refs [29,30,31] for health professionals' salary and laboratory services cost.

^3^This result is calculated according to the average length of stay × number of clients hospitalized × 433.85$/day, see ref [30]. If the duration of all hospitalizations is known, the TDH is used directly to calculate the average cost of hospitalizations.

^4^For example, for the period 0–3 months before installation: the TDH is calculated by replacing unknown values with the known ALS (obtained from known values).

# of hospitalizations of unknown duration = 8; # of hospitalizations of known duration = 4, totalling 51 days;

Average length of stay (ALS) = 51 days/4 = 12.75 days, approx. 13 days;

TDH = known number of days + 8 (ALS) = 51 days + 8(13) = 155 days.

^5^salary: 33.54$/h, including benefits and payroll taxes. See ref [30].

### Utilization of health services and cost estimation

The number of home visits by care workers decreased after the second period of 3 months of telesurveillance use (Table 5), from 15.34 ± 20.09 visits per client to 10.37 ± 16.57 (p = 0.004). The decrease referred to all types of services: home assistance (280 to 186), social work (85 to 62) and occupational therapy/physiotherapy (48 to 13). This situation may be related to the fact that no deterioration was observed with physical and psychological indicators (Table 1). Cost of home care services decreased by 25.5% ($1,135 to $845) after the first 3 months and by 31.9% ($845 to $575) in the second 3 months period. Possibly because of the small number of hospitalizations, it was not possible to find a significant difference between the measurement times. The average length of stay per hospitalized client went down from 13 days (3 months prior to telesurveillance) to 9.5 (3 months after) and to 4 days (between the 4^th ^and 6^th ^month). The number of elders hospitalized for at least 24 hours decreased by 45% (11 to 6) after 3 months of using telesurveillance and remained at 7 clients in the second period of 3 months of use (see Table 5). The cost of hospitalizations dropped by 34.5% ($1,638 to $1,073) in the first 3 months and by 58.5% ($1,073 to $445) in the second 3-month period. The overall cost of health services per client decreased during the 6 months of using telesurveillance compared to the 3 months preceding its use. The total cost per client in health services went from $2,773 three months prior to telesurveillance use to $2,300 after the first 3 months of use and $1,402 in the second period of 3 months. This represents a total decrease in costs per client of 17% after 3 months and 39% in the second period of 3 months. To these costs must be added the $25/month that the clients had to pay for the call center service, which increased the total cost per client by $75 for each 3 months of use. Table 5 presents these costs.

## Discussion

Contrary to the positive effects noted in the literature [5,7,9-11], no significant improvement was observed in the elders' quality of life and life habits after using the telesurveillance service (objective 1). However, elders presented high scores that fell within the norms on the SF-12 and LIFE-H tests before receiving the telesurveillance service and the observation period was rather short.

The very positive data on overall satisfaction confirm the results in the literature [[6,13], Rooney, Studenski & Roman in [14,15,32]]. The negative comments made about the sensitivity and appearance of the buttons are similar to those reported by Davies and Muller [32] as reasons for not wearing the emergency button. In the present study, although the buttons were programmable, only a few users had asked for their sensitivity to be adjusted; the others did not receive any follow-up in this regard.

Significant positive impacts were observed for the caregivers, in terms of daily living support, well-being, and the burden of providing services to the patients. These data clearly confirm the literature [Gatz & Pearson in [6,7]]. However, there was noimpact on the caregiver's social life.

The ratio of 0.50 calls per client over six months to the call center for health events is three times higher than that reported by Montgomery [7]. This difference is probably attributable to the fact that nurses rather than non-health professional personnel answer the clients' questions about their health and medications. The percentage of accidental numbers is comparable to the rates reported by Davies and Muller [32]; the 50% drop in the incidence of accidental numbers during the second period of 3 months of use seems to indicate that an adjustment period is necessary. As for the lack of use of vocal reminders, this could be attributable to a number of factors: lack of knowledge of the "new" functions on the part of both professionals and elders, lack of client follow-up by professionals, and lack of ongoing training regarding utilization of the telephone by the researchers throughout the project period [33].

Finally, one can observe a 27% reduction in hospital stays per client after the first 3 months of telesurveillance use and a 58% drop in the second period of 3 months. These percentages are similar to those reported in the literature: 69%, 26% and 25.4% [9,34]. Hospital admissions did not decrease during the 6-month period, contrary to what is reported in the literature (48%, 26.4% and 59.2%). However, the number of clients hospitalized declined from 11 to 6. These results can be explained by the fact that fewer clients were hospitalized during the 6-month period but some were admitted to hospital more than once. However, these comparisons with the literature must be interpreted with caution because the data collected by call centers staffed by non- health professionals cover longer periods (1 to 3 years), relate to larger samples (between n = 100 and n = 1000) and are not specific to older adults at risk of falling. As for the clinical home care services provided by the local community health centers, they decreased by 29.4% in the second 3 months of telesurveillance use.

Cost evolution (at 0–3 months and 4–6 months after intervention) shows that registering older adults at a telesurveillance center staffed by nurses, upon a health professional recommendation, costs the health care system less than services provided without a telesurveillance system. Moreover, no negative effects on the well-being of the individuals and their families were reported. The 39% cost saving in the second 3 months is considerable, in terms of both hospitalizations and home care interventions. The economic data from this study corroborate Tinker's estimates [35] that in England it would cost the public system less to offer vulnerable older adults an emergency call service.

### Limits of the study

The short follow-up as well as the short intervention period (up to 6 months), the lack of an equivalent control group and the small sample size (n = 38) are the most important limitations of this study. Researchers had to deal with ethical concerns and financial constrains of working with three partners. The industry supported home equipment for a maximum of 50 clients. One of the community health care center supported financially health care professionals to recruit the elders following their regular practice and norms; nurses at the call center were especially dedicated for the new telesurveillance service but only for 9 months. The Canadian Institute of Health Research approved this project but gave financing support only for the research team. This tri-joint collaboration was necessary to realise the study. All of the limitations mentioned above were discussed with the partners, but, for ethical, methodological and financial concerns, it was not possible to eliminate them.

### Policy implications

Given the positive effect noted in this study that corroborates other works, it would be desirable for the telesurveillance service to be accessible to all older adults at risk of falling whose security at home is compromised. At the present time in Quebec, telesurveillance services with nurses are available in only three public health regions (on 16) at a out-of-pocket cost of CAN$25/month (semi-public service), while services with non-health professional personnel are available in all regions at a out-of-pocket cost of CAN$37/month (non public service). This low cost by health professionals versus non-health professional personnel is attributable to the public sponsorship. According to some professionals, the monthly cost is an obstacle to accessibility to the service for many elders [33]. It would be more effective and more economical for the health care system to absorb the monthly fee paid by the elder in order to facilitate access to the telesurveillance service by all vulnerable older adults.

## Conclusion

This study shows positive outcomes of a telesurveillance service staffed by nurses for older adults (quality of life, life habits, satisfaction with the service and the technology, caregivers' burden). The results also show that registering older adults at a telesurveillance center staffed by nurses, upon the recommendation of a health professional, costs the health care system less (thanks to decrease in hospitalizations and home care services), without negative impact on the well-being of the individuals and their family caregivers.

## Competing interests

The author(s) declare that they have no competing interests.

## Authors' contributions

CV conceived the design of the study, coordinated all the data collection and data analysis; organized all meetings regarding recruitment and research assistant formation; outlined and drafted the manuscript. DR conceived the design of the study, participated at the meeting with clinical partners for recruitment, coordinated the data management regarding the costs services and commented the manuscript. ID filled out questionnaires with the participants, managed the data base, performed the data analysis and commented the manuscript. MG filled out the questionnaires with the participants and commented the manuscript. LRT conceived the design of the study, participate at the meeting with clinical partners for recruitment and at the one for research assistant formation; and commented the manuscript. All authors read and approved the final manuscript.
